# Lower body functioning and correlates among older American Indians: The Cerebrovascular Disease and Its Consequences in American Indians Study

**DOI:** 10.1186/s12877-017-0697-8

**Published:** 2018-01-05

**Authors:** R. Turner Goins, Mark Schure, Paul N. Jensen, Astrid Suchy-Dicey, Lonnie Nelson, Steven P. Verney, Barbara V. Howard, Dedra Buchwald

**Affiliations:** 1 College of Health and Human Sciences, Western Carolina University, 3971 Little Savannah Road, Cullowhee, NC 28723 USA; 2 Community Health, Montana State University, 305 Herrick Hall, Bozeman, MT 59717 USA; 3 Department of Medicine, University of Washington, 1959 NE Pacific Street, Seattle, WA 98195 USA; 4 College of Medicine, Initiative for Research and Education to Advance Community Health, Washington State University, 1100 Olive Way, Suite 1200, Seattle, WA 98101 USA; 5 College of Nursing, Institute for Research and Education to Advance Community Health, Washington State University, 1100 Olive Way, Suite 1200, Seattle, WA 98101 USA; 6 Department of Psychology, University of New Mexico, Logan Hall, MSC03-2220, 1 University of New Mexico, Albuquerque, NM 87131-0001 USA; 7 MedStar Health Research Institute and Georgetown/Howard Universities Center for Clinical and Translational Sciences, 6525 Belcrest Road, Suite 700, Hyattsville, MD 20782 USA; 8 College of Medicine, Institute for Research and Education to Advance Community Health, Washington State University, 1100 Olive Way, Suite 1200, Seattle, WA 98101 USA

**Keywords:** American Indians, Lower body functioning, Short physical performance battery

## Abstract

**Background:**

More than six million American Indians live in the United States, and an estimated 1.6 million will be aged ≥65 years old by 2050 tripling in numbers since 2012. Physical functioning and related factors in this population are poorly understood. Our study aimed to assess lower body functioning and identify the prevalence and correlates of “good” functioning in a multi-tribe, community-based sample of older American Indians.

**Methods:**

Assessments used the Short Physical Performance Battery (SPPB). “Good” lower body functioning was defined as a total SPPB score of ≥10. Potential correlates included demographic characteristics, study site, anthropometrics, cognitive functioning, depressive symptomatology, grip strength, hypertension, diabetes mellitus, heart disease, prior stroke, smoking, alcohol use, and over-the-counter medication use for arthritis or pain. Data were collected between 2010 and 2013 by the Cerebrovascular Disease and Its Consequences in American Indians Study from community-dwelling adults aged ≥60 years (*n* = 818).

**Results:**

The sample’s mean age was 73 ± 5.9 years. After adjustment for age and study site, average SPPB scores were 7.0 (95% CI, 6.8, 7.3) in women and 7.8 (95% CI, 7.5, 8.2) in men. Only 25% of the sample were classified with “good” lower body functioning. When treating lower body functioning as a continuous measure and adjusting for age, gender, and study site, the correlates of better functioning that we identified were younger age, male gender, married status, higher levels of education, higher annual household income, Southern Plains study site, lower waist-hip ratio, better cognitive functioning, stronger grip strength, lower levels of depressive symptomatology, alcohol consumption, and the absence of hypertension, diabetes mellitus, and heart disease. In our fully adjusted models, correlates of “good” lower body functioning were younger age, higher annual household income, better cognitive functioning, stronger grip, and the absence of diabetes mellitus and heart disease.

**Conclusions:**

These results suggest that “good” lower body functioning is uncommon in this population, whereas its correlates are similar to those found in studies of other older adult populations. Future efforts should include the development or cultural tailoring of interventions to improve lower body functioning in older American Indians.

## Background

More than six million American Indians live in the United States with an estimated 600,000 who are aged ≥65 years old [1]. By 2060, the number of American Indians who are aged ≥65 years old will increase to 1.6 million, tripling in numbers since 2012 [2]. Physical functioning and related factors in this population are poorly understood. Limited data indicate that disability is more common in older American Indians than in older Whites. Disability is usually a manifestation of poor health, and is a risk factor for falls and further disability [3]. Disability also has robust positive associations with receipt of informal caregiving, hospitalization, nursing home placement, and mortality [4–6]. Two studies that examined the 2000 US Census data demonstrated that older American Indians experience higher rates of disability than their same-aged counterparts of other races and ethnicities [7, 8]. For instance, this research found that American Indians aged ≥55 years had higher prevalence of self-reported disability than same-aged Whites (functional limitation: 36% vs. 25%; mobility disability: 21% vs. 17%; and, self-care disability: 12% vs. 9%) [7]. The second study examined American Indians who were aged ≥65 years and the researchers found that 58% had a disability compared to 42% in all other racial/ethnic groups aged ≥65 years [8].

Compared to the widely used self-reported measures of functional limitation and disability, performance-based measures of physical functioning are less likely to be influenced by psychosocial factors of the patient or study participants’ perception of their ability to perform certain activities [9]. Moreover, studies have demonstrated weak to moderate correlations between self-reported and performance-based physical functioning measures [9–11]. Identified American Indian cultural and social factors [12, 13] may influence the accuracy of such measurements and thus making it important to capture functioning in this population with performance measurements.

Yet, to date, there has only been one other study has used such measures to examine physical functioning in this population [14]. Thus, our objectives are to characterize the prevalence of good lower body functioning and identify its correlates in a multi-tribe, community-based sample of older American Indians. Our current will serve as a confirmatory study as well offering an examination with older American Indians who reside in three different geographic regions and the opportunity to examine some additional potential correlates.

## Methods

### Data source

Our data were collected as part of the Cerebrovascular Disease and its Consequences in American Indians (CDCAI) Study also known as the Strong Heart Stroke Study. The CDCAI Study was a cross-sectional study of cerebrovascular disease conducted with 1033 Strong Heart Study surviving participants who were aged ≥60 years [15], but 215 of the participants were removed from analyses due to one tribal community that subsequently withdrew study consent. We recruited participants from three study locations, including the Northern Plains, the Southern Plains, and the Southwestern United States. Participants were recruited for the study with trained field staff making the initial contact by telephone or during a planned home visit. The study’s purpose was explained by the field staff who would then invite the individual to participate in the study. If the individual was willing, field staff would screen them for study eligibility. Study exclusion criteria included prior surgery for a cerebral aneurysm; an implanted cardiac pacemaker, defibrillator, or artificial heart; contraindicating metal prostheses; a cochlear implant, spinal cord stimulator, or other implanted electrical device(s); history as a metal worker given the possibility of retained metal fragments; body weight of ≥350 pounds; and/or physical or cognitive inability to complete study procedures. Between 2010 and 2013, all of the participants received clinical, cognitive, and functioning assessments. Study procedures were approved by 13 organizations that included five Tribal Review Boards or Tribal Councils, five academic or medical institutional review boards (IRBs), and three regional Indian Health Service IRBs. The primary institution that provided institutional review board approval for this study was the University of Washington. As stipulated in the tribal approvals, we are unable to identify the other approving entities for this study in order to maintain the anonymity of the participating tribes. The design and recruitment methods for the CDCAI Study are described in more detail elsewhere [16].

### Measures

#### Physical functioning

The Short Physical Performance Battery (SPPB) measured lower body functioning using standing balance, gait speed, and chair stands [17]. SPPB scores are associated with falls [18], disability [17, 19], nursing home admission [17], and mortality [17, 20]. The validity and reliability of the SPPB have been established in large, community-based, geographically and racially diverse samples of older adults [17, 21].

Standing balance was assessed by asking each participant to attempt three increasingly difficult positions without the use of assistance devices and to hold each position for 10 s. Participants first had their standing balance examined with feet side-by-side, then in a semi-tandem position with the heel of one foot beside the big toe of the other, and finally in a tandem position with the heel of one foot directly in front of the toes of the other. For the side-by-side position standing task, participants were scored as 1 if they held the position for 10 s and 0 if they did not attempt or were unable to hold the position for 10 s. For the semi-tandem position standing task, participants were scored as 1 if they held the position for 10 s and 0 if they did not attempt or were unable to hold the position for 10 s. For the tandem standing task, participants were scored as 2 if they held the position for 10 s, 1 if they held the position for 3–9.99 s, and 0 if they did not attempt or held the position for <3 s. The three standing balance positions were summed to generate a total balance score that ranged from 0 to 4.

For gait speed, participants were asked to walk a 15-ft straight course at their usual pace continuing beyond the end of the course if they felt they could do so safely. Participants were permitted to use a cane or other walking aid as needed. Scores for the 15-ft walk were adapted from the 3- and 4-m walks by using the same pace requirements and extrapolating time limits to account for the additional distance covered. Participants were scored as 0 if they were unable to perform the walk, 1 if they completed the walk in more than 9.94 s, 2 if they completed in ≤9.94 s, 3 if they completed in ≤7.09 s, and 4 if they completed in ≤5.52 s.

For chair stands, study site field staff determined whether each participant could safely stand up from sitting in a chair without assistance. Those who could were then asked to stand up from the chair five times as quickly as possible without using their arms. Participants were scored as 0 if they were unable to complete 5 chair stands or completed 5 chair stands in >60 s, 1 if they completed in 16.7–60.0 s, 2 if they completed in 13.7–16.69 s, 3 if they completed in 11.2–13.69 s, and 4 if they completed in <11.2 s.

All SPPB task scores range from 0 to 4 and the total SPPB score is the sum of all three task scores, which ranges from 0 to 12 where higher scores are reflect better performance. We examined the individual SPPB tasks and total score as continuous measures as well as examined the total SPPB score as a binary measure such that ≥10 denoted “good” performance and ≤9 denoted “poor” performance [22].

#### Independent variables

Independent variables included demographic characteristics, study site, anthropometrics, cognitive functioning, depressive symptomatology, grip strength, hypertension, diabetes mellitus, heart disease, prior stroke, smoking, alcohol use, and over-the-counter arthritis or pain medication use. Demographic characteristics included age, gender, marital status, educational attainment, and annual household income. Anthropometrics included body mass index (BMI) and waist-hip ratio, which were directly measured during CDCAI assessments. BMI was calculated as measured weight in kilograms divided by measured height in meters squared; waist circumference was measured at the umbilicus with the participant in a supine position; and hip circumference was measured at the widest portion of the buttocks with the participant standing.

Cognitive functioning was measured with the Modified Mini-Mental State (3MS) examination, which has possible scores ranging from 0 to 100 where higher scores are reflective of better cognitive functioning. The 3MS includes screening items on temporal and spatial orientation, immediate and delayed memory, attention and concentration, language and naming, verbal fluency, and executive functioning [23]. Depressive symptomatology was measured with the Centers for Epidemiologic Studies Depression Scale (CES-D) [24], a 20-item instrument that describes the frequency of symptoms within the last week by using a 4-point scale ranging from 0 (rarely or none of the time) to 3 (most or all of the time). When scored, the CES-D ranges from 0 to 60, with higher scores indicating more depressive symptomatology. Scores were analyzed as a binary variable by using the standard cutoff score of ≥16 to reflect a clinically significant level of symptoms [24].

Grip strength was ascertained three times in kilograms for both hands by using a calibrated dynamometer. Measures of the participant’s dominant hand were averaged for use in our analyses. Hypertension was assessed with measured blood pressure (systolic ≥140 or diastolic ≥90) or self-reported use of antihypertensive medication. Diabetes mellitus was defined as fasting glucose of at least 126 mg/dL or self-reported use of insulin or an oral hypoglycemic. Heart disease was determined with a “yes” answer to the question, “Has a medical person ever told you that you have (or had): congestive heart failure, a heart attack, any other heart trouble, a bypass, a valvular repair or replacement, and/or a pacemaker installed?” Stroke was also determined with a “yes” answer to the question, “Has a medical person ever told you that you have had a stroke?”

Participants were asked about current tobacco smoking and alcohol consumption in the past 30 days, with “yes” or “no” response options. Lastly, over-the-counter medication use for arthritis or pain was determined with a “yes” answer to the question, “Do you take over-the-counter medicines for arthritis or pain, like Advil, Motrin, or Aleve?”

#### Statistical analyses

We used mean, standard deviation (SD), count, and percent to describe participant characteristics, including total SPPB scores and scores on the three individual SPPB tasks. To assess associations of demographic characteristics, anthropometrics, cognitive functioning, depressive symptoms, grip strength, clinical conditions, lifestyle behaviors, and over-the-counter medication use for arthritis or pain with both individual SPPB task and total SPPB scores, linear regression models were used with adjustment for age, gender, and study site. Our result estimates were reported using 95% confidence intervals (95% CI) for each independent factor individually. Estimates for continuous independent variables were given per clinically meaningful change in unit, which tended to be close to the estimate of standard deviation for the entire sample population. To assess associations between selected independent variables and the binary total SPPB score (good versus poor), generalized linear models with Poisson distribution and log-link were used, with inclusion of all independent factors in the same model. Continuous variables were transformed based on their standard deviations in order to facilitate direct comparison of degree of effect from each of the different independent variables. Exponentiated Poisson coefficients were interpreted as relative risk under assumption of reasonably rare outcome (25%). All of our analyses were performed with Stata 13 [25].

## Results

Among 818 CDCAI Study participants, two did not complete the SPPB and were excluded. from our analyses, yielding an analytic sample size of 816. The mean age of the sample was 73 ± 5.9 years with an age range of 64 to 95 years. After adjustment for age and study site, average SPPB scores were 7.0 (95% CI, 6.8, 7.3) in women and 7.8 (95% CI, 7.5, 8.2) in men, and scores for the balance, gait, and chair stand tasks declined with increasing age. Twenty-five percent of the study population was classified with “good” lower body functioning. By gender, 20% of women and 36% of men had “good” lower body functioning and by study site, 44% in the Northern Plains, 46% in the Southern Plains, and 10% in the Southwest had “good” lower body functioning (data not shown). Characteristics of this sample are reported in Table 1 by women and men.

**Table 1:** Selected characteristics of participants from the Cerebrovascular Disease and its Consequences in American Indians Study

	Women (*n* = 554)	Men (*n* = 262)
Age, mean (SD)	73 (6.1)	73 (5.3)
Study site		
Northern Plains	46%	45%
Southern Plains	40%	46%
Southwest	13%	9%
Married	29%	57%
Highest education		
Less than high school	21%	18%
High school graduate	25%	26%
College or greater	54%	56%
Annual household income		
< $25 k	66%	49%
$25 k–50 k	28%	36%
≥ $50 k	7%	15%
BMI (kg/m^2^)		
≤ 24.9	17%	12%
25–29.9	29%	31%
≥ 30	54%	57%
Waist-hip ratio, mean (SD)	0.9 (0.1)	1.0 (0.1)
3MS score, mean (SD)	88 (9.6)	88 (10.0)
CESD score, mean (SD)	11 (8.5)	10 (7.0)
CESD ≥16	24%	18%
Grip strength (kg), mean (SD)	20 (6.2)	34 (8.7)
Hypertension	81%	81%
Diabetes mellitus	50%	48%
Heart disease	27%	36%
Stroke	9%	8%
Current smoker	19%	25%
Alcohol consumption in past 30 days	8%	26%
OTC pain medication use	38%	27%

Values given as % unless otherwise specified. *SPPB* Short Physical Performance Battery, *IQR* Interquartile range, *SD* Standard deviation, *BMI* Body mass index, *3MSE* Modified Mini-Mental State Examination, *CES-D* Centers for Epidemiological Studies depression scale, *OTC* Over the counter

Table 2 presents results from linear regression models in which we examined associations of independent variables with individual task and total SPPB scores. In a limited model that included terms only for age, gender, and study site, older age was significantly associated with lower individual task and total SPPB scores, with nearly a full point differential in total SPPB for each 5 additional years of age (β = −0.91, 95% CI: -1.07, −0.74). Male gender was significantly associated with higher gait and chair stand task and total SPPB scores compared with women (β = 0.78, 95% CI: 0.37, 1.19). Compared with Northern Plains, participants in the Southern Plains on average had better balance and total SPPB scores but no difference in gait speed or chair stands. There was no evidence of difference between Northern Plains and Southwest participants. We also found that being married, having higher levels of educational attainment, having higher annual household income, having lower waist-hip ratio, and having better grip strength were significantly associated with higher individual task and total SPPB scores, with total SPPB score most strongly affected. Higher (better) 3MS score was significantly associated with higher individual task and total SPPB scores, with more than 1 point in total SPPB associated with each additional 10 points in 3MS (β = 1.08, 95% CI: 0.88, 1.27). More depressive symptoms was significantly associated with lower task and total SPPB scores; dichotomized clinical depression (CES-D ≥ 16) was similarly associated, with depressed participants scoring, on average, 1.24 points lower on total SPPB (95% CI: -1.70, −0.79). Prevalent hypertension, diabetes mellitus, heart disease, and prior stroke were all significantly associated with lower individual task and total SPPB scores. Alcohol consumption was associated with a higher gait score while current smoking and use of over-the-counter arthritis or pain medications were not associated with individual task or total SPPB scores.

**Table 2:** Multivariate linear regression models of the Short Physical Performance Battery (SPPB) score among participants from the Cerebrovascular Disease and its Consequences in American Indians Study

	Balance score		Gait score		Chair Stand score		Total score	
	β	95% CI	β	95% CI	β	95% CI	β	95% CI
Age (per 5 years)	−0.31	(−0.38, −0.23)	−0.31	(−0.37, −0.25)	−0.29	(−0.36, −0.22)	−0.91	(−1.07, −0.74)
Male gender	0.12	(−0.07, 0.31)	0.42	(0.26, 0.57)	0.25	(0.07, 0.42)	0.78	(0.37, 1.19)
Study site								
Northern Plains	*Reference*		*Reference*		*Reference*		*Reference*	
Southern Plains	0.31	(0.13, 0.50)	0.01	(−0.15, 0.16)	0.09	(−0.09, 0.26)	0.41	(0.00, 0.82)
Southwest	−0.15	(−0.43, 0.14)	−0.13	(−0.37, 0.11)	−0.06	(−0.33, 0.21)	−0.33	(−0.96, 0.29)
Married	0.29	(0.10, 0.48)	0.25	(0.10, 0.41)	0.39	(0.21, 0.57)	0.93	(0.52, 1.34)
Educational attainment								
Less than high school diploma	*Reference*		*Reference*		*Reference*		*Reference*	
High school graduate	0.45	(0.19, 0.71)	0.37	(0.16, 0.58)	0.36	(0.12, 0.61)	1.19	(0.62, 1.75)
Some college or greater	0.56	(0.32, 0.79)	0.58	(0.39, 0.78)	0.44	(0.21, 0.66)	1.58	(1.05, 2.10)
Annual household income								
< $25,000	*Reference*		*Reference*		*Reference*		*Reference*	
$25,000–50,000	0.31	(0.11, 0.51)	0.37	(0.20, 0.53)	0.38	(0.19, 0.56)	1.05	(0.62, 1.48)
≥ $50,000	0.43	(0.12, 0.75)	0.45	(0.19, 0.71)	0.69	(0.40, 0.98)	1.57	(0.89, 2.25)
BMI								
≤ 24.9 kg/m2	*Reference*		*Reference*		*Reference*		*Reference*	
25–29.9 kg/m2	−0.12	(−0.40, 0.15)	−0.11	(−0.34, 0.12)	−0.04	(−0.30, 0.23)	−0.27	(−0.87, 0.34)
≥ 30 kg/m2	−0.11	(−0.37, 0.15)	−0.03	(−0.25, 0.18)	−0.10	(−0.35, 0.14)	−0.24	(−0.81, 0.32)
Waist-hip ratio (per 0.1)	−0.27	(−0.41, −0.13)	−0.20	(−0.32, −0.08)	−0.24	(−0.38, −0.11)	−0.71	(−1.02, −0.41)
3MS score (per 10 points)	0.42	(0.33, 0.51)	0.38	(0.30, 0.45)	0.28	(0.19, 0.36)	1.08	(0.88, 1.27)
CES-D score (per 8 points)	−0.20	(−0.28, −0.11)	−0.17	(−0.24, −0.10)	−0.22	(−0.30, −0.14)	−0.59	(−0.77, −0.40)
Depression (CES-D ≥ 16)	−0.42	(−0.63, −0.21)	−0.32	(−0.50, −0.15)	−0.50	(−0.69, −0.30)	−1.24	(−1.70, −0.79)
Grip strength (per 6 kg)	0.30	(0.23, 0.38)	0.26	(0.20, 0.33)	0.25	(0.18, 0.32)	0.82	(0.66, 0.97)
Hypertension	−0.38	(−0.60, −0.16)	−0.27	(−0.45, −0.08)	−0.24	(−0.45, −0.03)	−0.89	(−1.37, −0.40)
Diabetes mellitus	−0.60	(−0.77, −0.42)	−0.40	(−0.54, −0.26)	−0.54	(−0.70, −0.38)	−1.54	(−1.91, −1.16)
Heart disease	−0.38	(−0.57, −0.19)	−0.25	(−0.41, −0.09)	−0.40	(−0.58, −0.23)	−1.03	(−1.45, −0.62)
Self-reported prior stroke	−0.62	(−0.94, −0.31)	−0.33	(−0.59, −0.07)	−0.36	(−0.65, −0.07)	−1.32	(−2.00, −0.63)
Current smoker	−0.14	(−0.36, 0.07)	0.13	(−0.05, 0.31)	−0.06	(−0.26, 0.15)	−0.08	(−0.56, 0.40)
Alcohol consumption in last 30 days	0.03	(−0.23, 0.29)	0.24	(0.02, 0.45)	0.15	(−0.10, 0.40)	0.41	(−0.16, 0.99)
Use OTC pain medications	−0.06	(−0.25, 0.12)	0.00	(−0.16, 0.15)	−0.08	(−0.25, 0.09)	−0.15	(−0.55, 0.26)

Each line represents a separate model. All models adjusted for age category, gender, and study site. β = beta coefficient; *95% CI* 95% confidence interval, *BMI* Body mass index; *3MSE* Modified Mini-Mental State Examination, *CES-D* Centers for Epidemiological Studies depression scale, *OTC* Over the counter

**Fig. 1:**
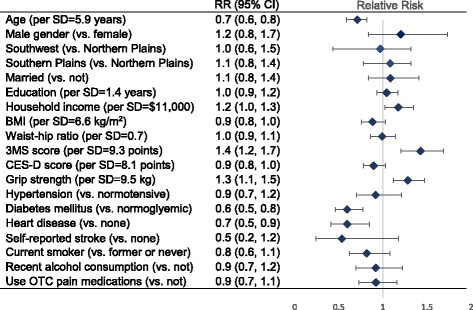
Estimates of relative risk (RR) with 95% confidence intervals (95% CI) for independent factors in association with good lower body functioning (total short physical performance battery, SPPB scores ≥10, compared with poor functioning, or scores <10) from a multivariate, mutually-adjusted generalized Poisson model with log-link and robust standard errors. BMI = Body mass index; 3MSE = Modified Mini-Mental State Examination; CES-D = Centers for Epidemiological Studies depression scale; OTC = Over the counter

To directly compare factors in terms of individual factors’ influence on lower body functioning, limited generalized regression models for the dichotomized total SPPB score, with mutual adjustment for all independent terms, detected significant associations for age, annual household income, cognitive functioning, grip strength, diabetes mellitus, and heart disease (Fig. 1). Each standard deviation increase in age was associated with 30% decrease in likelihood of “good” function (95% CI: 20–40%); similarly, presence of diabetes mellitus and heart disease were associated with 40% and 30% decreased likelihood of “good” lower body function, respectively (95% CIs: 20–50%; 10–50%). Conversely, each SD in annual household income—corresponding to $11,000—was associated with 20% increased likelihood of “good” lower body function (95% CI: 0–30%). Grip strength and 3MS score were similarly positively associated, with each SD increase corresponding to 30% and 40% different likelihood of “good” function, respectively (95% CIs:10–50%; 20–70%).

Male gender and married status were associated with better function; higher BMI, more depressive symptoms, presence of hypertension, prior stroke, smoking, alcohol consumption, or OTC pain medications were associated with worse function. However, none of these associations excluded the possible role of chance, once adjusted for all other factors. Educational attainment and waist-hip ratio were not associated with lower body functioning. Lastly, there was no evidence of differences in SPPB by site, after adjustment for all other factors.

## Discussion

Our findings suggest that “good” lower body functioning was relatively rare in this cohort and varied by study site. Overall lower body functioning was poor in most men and women, although it was worse in women. When treating lower body functioning as a continuous measure and adjusting for age, gender and study site, the correlates of better functioning that we identified were younger age, male gender, married status, higher levels of education, higher annual household income, Southern Plains study site, lower waist-hip ratio, better cognitive functioning, stronger grip strength, lower levels of depressive symptomatology, alcohol consumption, and better cardiovascular health. When measuring lower body functioning as a binary outcome and adjusting for age, gender, study site and the remaining covariates, the correlates of “good” lower body functioning that we identified were younger age, higher annual household income, better cognitive functioning, stronger grip, and the absence of diabetes mellitus and heart disease.

Notably, the CDCAI Study cohort had a substantially lower prevalence of “good” lower body functioning than the sample of community-dwelling American Indians aged ≥55 years recruited for the Native Elder Care Study. In that sample, 48% of participants had SPPB scores of ≥10 [14] while only 25% of the CDCAI sample had total SPPB scores of ≥10. Since the CDCAI cohort is slightly older, it is unclear whether these differences in functioning derive primarily from differences in age. Studies using SPPB have also demonstrated lower levels of physical functioning in other racial and ethnic minority populations compared to same-aged Whites. For example, one recent study found that older African American men had lower SPPB scores than older White men after adjusting for age, rural residence, marital status, education, and income [26].

The correlates of better or “good” lower body functioning identified in our study are consistent with those identified by the Native Elder Care Study. Both studies identified higher socioeconomic status, fewer comorbid conditions, and less depressive symptomatology to be associated with better functioning while BMI and smoking status were unrelated with functioning [14]. No relationship between BMI and lower body functioning has been demonstrated in older American Indians. The undetected association between BMI and lower body functioning is not entirely surprising given that prior studies have not presented a consistent finding [27–29]. However, when measuring functioning with self-reported activities of daily living, the research appears to be clearer in demonstrating that overweight is associated with poorer functioning [30]. Our study is the first study to examine the association of waist-hip ratio with lower body functioning in older American Indians. Other research with older adults of other race and ethnicities have generally found a consistent significant relationship between higher waist-hip ratios with poorer lower body functioning [29, 31, 32]. Since BMI does not distinguish between muscle and fat and waist-hip ratio captures central adiposity, waist-hip ratio is considered a better measure when assessing correlates or predictors of lower body functioning. In light of our findings, future research would need to assess more closely the relative contribution and mechanism of influence of BMI versus waist-hip ratio on lower body functioning in older American Indians. Additional research may also examine in greater detail the observed differences in degree of associations between health measures with individual SPPB tasks to illuminate different mechanisms of biological effect. For example, such research may want to examine why cognitive functioning had a stronger association with balance and gait than with chair stands.

The present study has several strengths, including a population-based design and the use of a large, multi-tribe sample derived from three geographically distinct regions including the Northern Plains, Southern Plains, and the Southwest. Notably, the tribes in each region are also culturally and historically distinct from those in the Southeast as was examined in the Native Elder Care Study. Additional strengths of this study include the use of objective measures and the comprehensive inclusion of potential correlates. Objective measurement of lower body functioning is particularly important in American Indian geriatric research, given the potential effects that cultural factors may have on the validity of self-report physical functioning measures in this population. Previous work has identified constructs of “tolerated illness” and the “harmony ethic” in American Indian cultures, such that the ability to endure hardships and poor health without imposing one’s needs on others is valued [12, 13].

Despite these strengths, certain limitations of our study must also be noted. First, all study participants were older adults who were survivors from the Strong Heart Study cohort and generalizing our findings may not extend to other groups. Second, our study data were cross-sectional, precluding our ability to directly examine temporal sequence in the reported associations. Third, cognitive measures such as the 3MS have not been validated or normed with American Indians making the clinical relevance of changes in the score unclear. Future research is needed that would identify appropriate normative standards in this measure as well as any necessary cultural adaptations. Also, study exclusion criteria limited participation to people who could travel to complete the study’s complex medical, cognitive, and functional assessments. This factor might have artificially inflated our estimates of “good” lower body functioning. Last, the examination of a large number of covariates resulted in multiple comparisons, so some associations may have appeared due to chance. However, the directionality of these associations with “good” lower body functioning were similar to those reported in an independent sample of American Indians [14], suggesting it is unlike that these associations are spurious.

## Conclusions

The distinct circumstances of our study population that contribute to this high prevalence of poor physical functioning must be better understood. The literature on American Indian health continues to find higher prevalence of many chronic illnesses than in the all-races or White populations [33, 34], while older American Indians have lower educational attainment and income compared to their same-aged peers [35]. A better understanding of current physical functioning in older members of this population can be gained through a life course approach that follows people from birth to old age [36]. All racial and ethnic minority populations in the U.S. have an experience of life that differs markedly from that of Whites because of social and structural inequalities. Given their unique history, American Indians have endured hardships comparable to, or even more severe than, those in other populations. While interventional work has been conducted to improve mobility among older adults, these interventions have not been implemented in tribal communities [37–39]. Subsequent efforts should include translating such promising efforts for Indian Country with the goal of creating sustained community-based interventions that promote mobility and overall good physical health.
